# Evaluation of Xpert *MTB-RIF* guided diagnosis and treatment of rifampicin-resistant tuberculosis in Indonesia: A retrospective cohort study

**DOI:** 10.1371/journal.pone.0213017

**Published:** 2019-02-28

**Authors:** Arto Yuwono Soeroto, Bony Wiem Lestari, Prayudi Santoso, Lidya Chaidir, Basti Andriyoko, Bachti Alisjahbana, Reinout van Crevel, Philip C. Hill

**Affiliations:** 1 Department of Internal Medicine, Respirology and Critical Illness Division, Faculty of Medicine Universitas Padjadjaran, Hasan Sadikin General Hospital, Bandung, Indonesia; 2 Department of Public Health, Faculty of Medicine Universitas Padjadjaran, Bandung, Indonesia; 3 TB-HIV Research Center, Faculty of Medicine Universitas Padjadjaran, Bandung, Indonesia; 4 Department of Internal Medicine, Radboud Institute for Health Sciences, Radboud University Medical Center, Nijmegen, The Netherlands; 5 Department of Clinical Pathology, Faculty of Medicine Universitas Padjadjaran, Hasan Sadikin General Hospital, Bandung, Indonesia; 6 Centre for Tropical Medicine and Global Health, Nuffield Department of Medicine, University of Oxford, Oxford, United Kingdom; 7 Center for International Health, University of Otago, Dunedin, New Zealand; University of Cape Town, SOUTH AFRICA

## Abstract

**Background:**

Rifampicin-resistant tuberculosis (RR-TB) is largely underdetected in Indonesia. Xpert MTB/RIF (Xpert) has recently been introduced, prioritizing patients at risk of RR-TB, followed by phenotypic drug-susceptibility (DST) if rifampicin resistance is detected.

**Objective:**

This study investigated Xpert-based management of presumptive RR-TB cases under routine practice in West Java, Indonesia.

**Methods:**

We examined all records of patients tested with Xpert in the referral hospital for West Java in 2015–2016. We measured loss across a limited cascade of care, time to Xpert diagnosis and the commencement of initial second-line treatment, and identified factors associated with diagnostic and treatment delay. Additionally, we analyzed the appropriateness of treatment according to DST results.

**Results:**

Of 3415 patients with presumptive RR-TB, 3215 (94%) were tested by Xpert, of whom 339 (10.5%) were diagnosed as RR-TB. 288 (85%) of 339 RR-TB patients started initial second-line TB treatment, with 48 (14%) patients being lost between diagnosis and pre-treatment assessment. Second-line treatment was commenced at a median of 41 days (IQR 29–70) after RR-TB diagnosis. Delays in both diagnosis and treatment initiation were observed in 104 (52%) of 201 RR-TB patients with identifiable referral date. Rural residence was associated with delay to diagnosis (adjusted OR 2.7; 95%CI 1.5–5.2) and treatment initiation (adjusted OR 2.0; 1.2–3.4). Of 162 patients with available DST result, 107 (66%) had multidrug-resistant tuberculosis (MDR-TB) and 32 (20%) had either pre-extensively drug resistant (pre-XDR) or extensively drug resistant tuberculosis (XDR-TB). We estimated that with the current algorithm 41% of pre-XDR or XDR-TB patients are diagnosed, and 33% of them started on an appropriate treatment regimen.

**Conclusions:**

Many patients with Xpert-diagnosed RR-TB either do not start MDR-TB treatment or encountered diagnostic and treatment delays under programmatic conditions in Indonesia, and most pre-XDR and XDR-TB cases remain undiagnosed. Further expansion and ongoing quality improvement of RR-TB services are urgently needed.

## Introduction

The emergence of rifampicin-resistant tuberculosis (RR-TB) has become a threat to global TB control. Suboptimal TB treatment, poor treatment outcomes, low adherence to TB treatment, and poorly regulated private sectors have contributed to the increase of MDR-TB [1, 2]. Most incident of RR-TB cases are thought to result from direct transmission rather than treatment-related resistance acquisition [3], like in South Africa where 52% of RR-TB is caused by direct transmission [4]. Thus, improving RR-TB diagnosis should be a high priority. Xpert MTB/RIF assay (Xpert) allows for universal limited drug-susceptibility testing (DST) with rapid detection of RR-TB [5]. The World Health Organization (WHO) recommends Xpert as an initial diagnostic tool for presumptive RR-TB patients [6]. Since 2011, the global roll-out of Xpert has increased detection of RR-TB diagnosis three- to eight-fold compared to conventional testing [7].

Indonesia has the second highest number of TB cases globally, with an estimated TB prevalence of 647/100,000, and it is also considered a high burden RR-TB country [8]. The latest drug-resistance survey in Indonesia reported RR-TB in 2% of new cases and 9.7% of re-treatment cases [9]. The Indonesian National TB Programme (NTP) has adopted Xpert as a frontline test to detect RR-TB since 2012, yet only a limited number of healthcare facilities have access to Xpert and many patients have restricted access to second-line TB treatment [10]. As a result, RR-TB very often remains undetected and untreated, leading to further spread of drug resistance, worse TB treatment outcomes and increased mortality [11].

History of multiple previous TB treatment, younger age, and irregular treatment, HIV infection [12], and diabetes [13] have been reported risk factors for RR-TB acquisition [1, 14–16], whereas previous TB treatment, resistance to ofloxacin, being smear-positive at the start of treatment, and no culture conversion by the 3rd month of treatment have been associated with poor MDR-TB treatment outcomes [17–19]. Various studies have reported diagnosis and treatment outcomes of RR-TB using Xpert as rapid DST [20–24]. Several studies have analysed the cascade of care for RR-TB under programmatic conditions [25–29], however, none have been performed in Indonesia. A cascade of care study from India’s public sector showed that a substantial proportion of MDR-TB patients who reached the NTP diagnostic facilities were not successfully diagnosed [25]. Therefore, we aimed to evaluate Xpert-based management of presumptive RR-TB cases under routine programmatic conditions in West Java, Indonesia, focusing on loss across a limited cascade of care from patient’s registration at Xpert facility to treatment initiation, diagnostic and treatment delay, and the identification of associated risk factors.

## Materials and methods

### Study setting

We performed a retrospective cohort study of adults with presumptive RR-TB who were registered for Xpert testing between 1 January 2015 and 31 December 2016 at the ‘programmatic management of drug-resistant tuberculosis (PMDT)’ clinic of Dr. Hasan Sadikin Hospital, the main referral site for patients with RR-, MDR-, pre-XDR- and XDR-TB in West Java (population: 46.4 million). In West Java, at the time of data collection, there were four hospitals with Xpert and only two that provided PMDT service, as authorized by the Ministry of Health. We excluded patients with a previous history of RR-TB treatment because their referral pattern is different from the presumptive RR-TB cases in our health system as they can directly seek care to PMDT clinic.

A presumptive RR-TB case was defined as an individual who has one or more risk factors for RR-TB according to Indonesian PMDT guidelines [9] (S1 Table) and registered at the PMDT clinic. With respect to an initial treatment regimen, MDR-TB is defined pragmatically as TB with rifampicin-resistance based on Xpert result. Patient with RR-TB detected by Xpert are treated empirically with a standard MDR-TB regimen while awaiting complete phenotypic DST results. Standard MDR-TB treatment in Indonesia consists of Kanamycin (Km) injections for a minimum eight months as initial phase and ethionamide (Eto) or prothionamide, levofloxacin (Lfx) or moxifloxacin, cycloserine (Cs) or p-aminosalicylic acid, pyrazinamide (Z) and ethambutol (E) (if no resistance was detected) for at least 20 months. When DST results indicate additional resistance, either pre-XDR or XDR-TB, the clinical expert team adjusts the treatment regimen accordingly, following the national PMDT guidelines [9]. Sputum culture and DST for Dr. Hasan Sadikin Hospital are routinely performed at the provincial reference laboratory in Bandung. Culture was done using solid or liquid media (MGIT). DST assesses resistance to first-line drugs (rifampicin, isoniazid, ethambutol, and streptomycin), and a limited number of second-line drugs (kanamycin, amikacin, ofloxacin).

### Outcome measures

**Fig 1:**
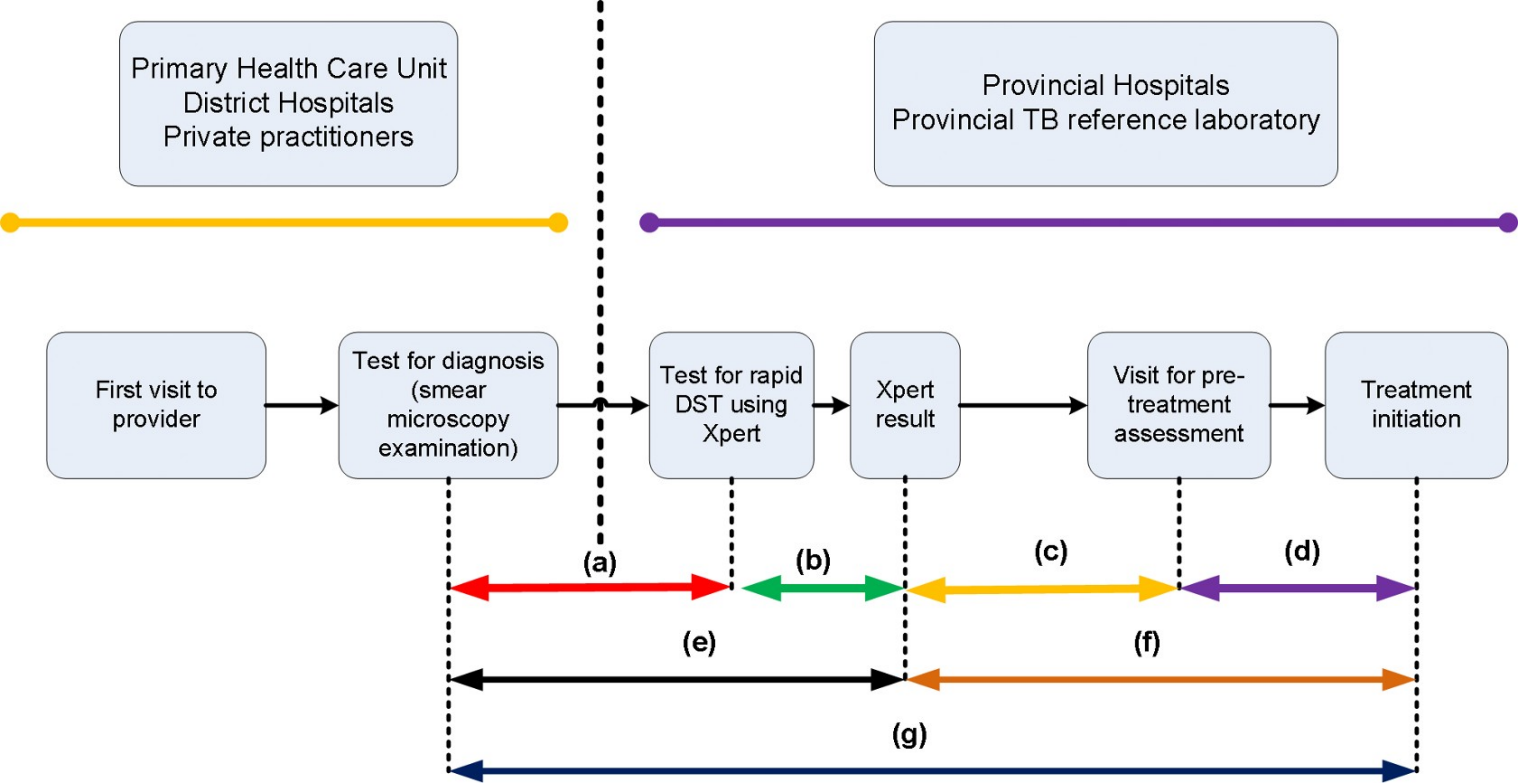
Time to diagnosis and treatment of presumptive rifampicin-resistant TB using Xpert. Time between microscopic TB diagnosis and patient presentation for Xpert examination (a), Xpert result (b), time between Xpert result and patients visit for pre-treatment assessment (c), initiation of second line treatment (d), total time between microscopic TB diagnosis and diagnosis of RR-TB (e), and total time between RR-TB diagnosis and start of second line treatment (f). Total time between microscopic TB diagnosis and start of MDR-TB treatment (g).

We used the conceptual framework described in Fig 1. We measured time between initial smear microscopy and the Xpert result as time to RR-TB diagnosis, and defined diagnostic delay as more than 14 days, on the basis of the median recorded time in our data. We defined initial treatment delay as more than 15 days between the Xpert result confirming RR-TB and the start date of MDR-TB treatment, in line with a previous study [30].

### Data collection and analysis

Presumptive RR-TB patients were identified from the clients’ register. We extracted data from the routine clinic staff assessment of patients, using a structured data collection tool gathering information about TB symptoms, sociodemographic characteristics, referral origin, risk factors for RR-TB, drug-sensitive TB (DS-TB) treatment history, and history of seeking care from private practitioners. We accessed laboratory results electronically through data linkage by matching patients’ unique identification numbers. The times between smear microscopic TB diagnosis (mostly at other TB clinics), phenotypic DST results, and between Xpert testing and second-line treatment were extracted from medical records and the electronic register. Appropriateness of second-line treatment was determined by the clinical expert team by assessing the DST results, patient TB treatment history and clinical symptoms [31].

We classified the type of residence according to whether the patients lived in or outside Bandung. The dates of smear microscopy results were obtained from referral letters from lower level healthcare providers. The date of the Xpert result was taken from the laboratory register at the hospital. We obtained the date of pre-treatment assessment and second line treatment initiation from the medical records.

Data were analyzed using IBM SPSS Statistics version 20. We used a simple extrapolation to estimate overall Xpert positivity and overall proportions of MDR-, pre-XDR- and XDR-TB for a cascade of care analysis. We plotted Kaplan Meier survival curves to illustrate both times to cascade step completion and proportion completing the step. Those lost-to-follow up were right censored at 180 days for each step. We conducted multivariable logistic regression to analyze risk factors associated with diagnosis and treatment delay using backward-stepwise method. The multivariable models included gender, age, type of residence, income, presumptive MDR-TB criteria, and frequency of previous DS-TB treatment as covariates. In the multivariable analyses, a p-value < 0.05 was considered statistically significant, and the adjusted odds ratio (AOR) and 95% confidence intervals (CI) were reported. Ethical clearance for this study was provided by the Health Research Ethics Committee at Faculty of Medicine, Universitas Padjadjaran under number 712/UN6.C2.1.2/KEPK/PN/2014. Informed consent was waived by the Ethics Committee as this was a retrospective analysis of secondary data. Further, the data from patient records were de-identified and analyzed anonymously.

## Results

### Xpert testing and MDR-TB treatment

**Fig 2:**
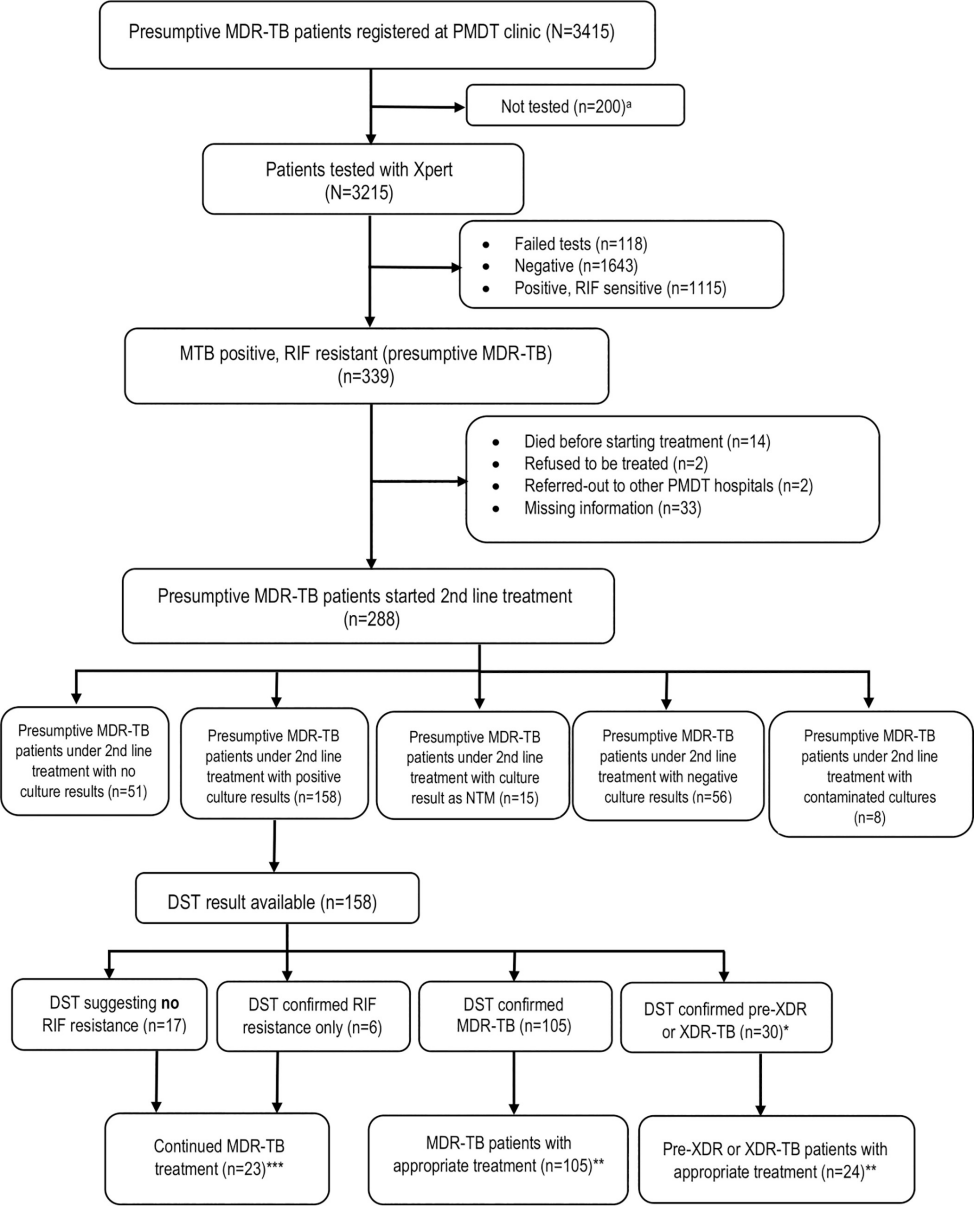
Flow chart of study participants. MDR-TB: Multidrug-resistant TB, PMDT: Programmatic Management of Drug-resistant TB, NTM: Non-tuberculous Mycobacteria, DST: phenotypic Drug Susceptibility Testing. *pre-XDR (n = 27) and XDR (n = 3). ** Appropriate treatment was defined as compatibility between DST result and the choice of drug-resistant treatment regimen. ***Patients with DST result not suggesting MDR continued their 2nd line treatment according to Indonesian PMDT guideline. ^a^The 200 patients were not tested due to possible reasons, which were: 1) patients didn’t present to the laboratory for Xpert testing; 2) patients weren’t able to expectorate sputum.

**Fig 3:**
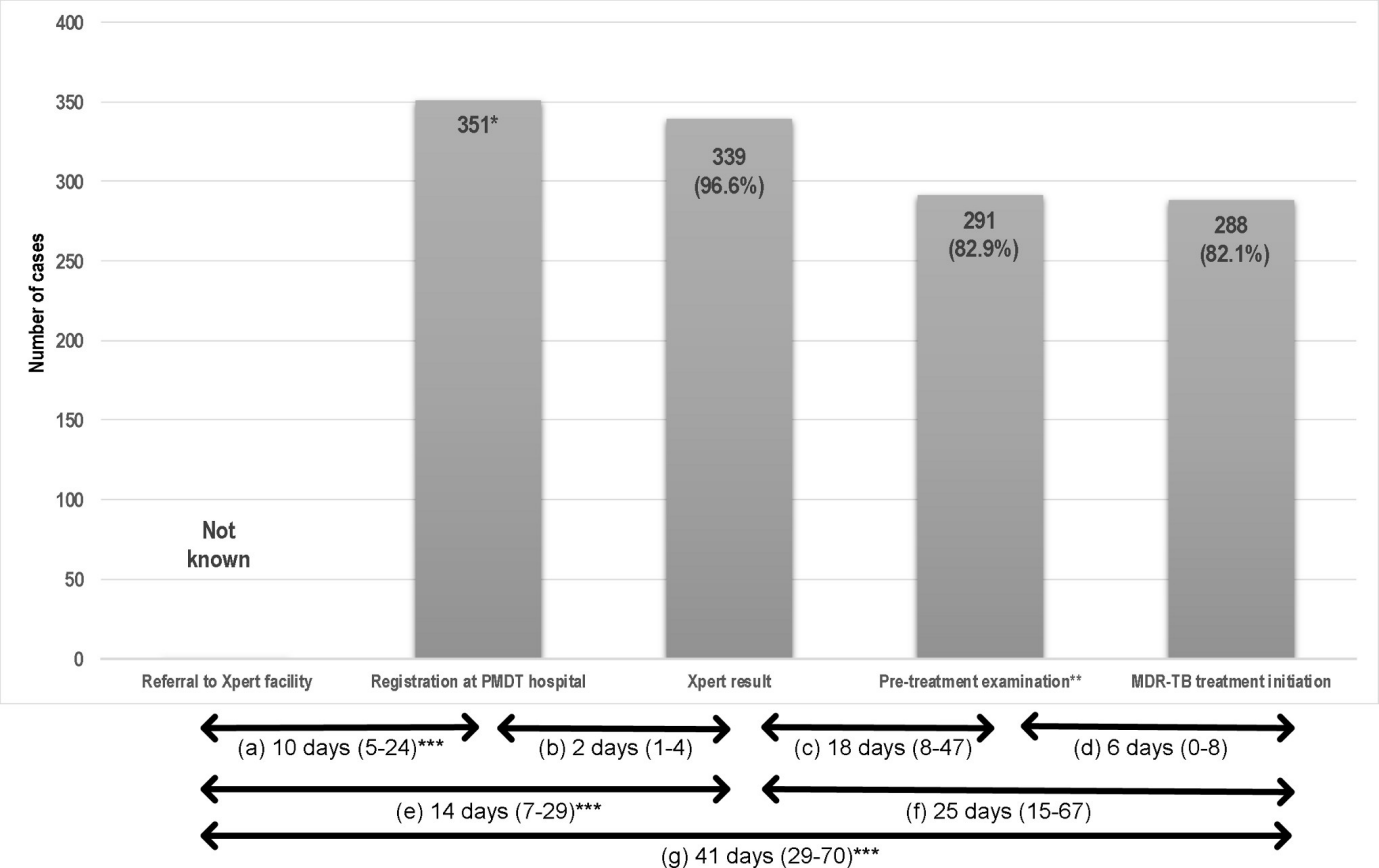
Cascade of care and time to diagnosis and treatment of Xpert rifampicin-resistant TB patients. Values in the bars indicate number (percentages). *12 of 118 with a failed result were imputed as Xpert positive and assigned the censored time of 180 days. **Pre-treatment examination included blood & urine test, HIV test, chest X-ray, audiometry and psychiatric assessment. ***Data were only available for 201 patients. Median (IQR) times between: microscopic TB diagnosis and patient presentation for Xpert examination (a), Xpert examination and Xpert result (b), Xpert result and visit for pre-treatment assessment (c), pre-treatment assessment and initiation of second line treatment (d), microscopic TB diagnosis and diagnosis of RR-TB (e), Xpert result and initiation of second line treatment (f), microscopic TB diagnosis and initiation of second-line treatment (g).

From the clinic register, there were 3415 presumptive cases of RR-TB, of whom 3215 (94.1%) were tested with Xpert. The presumptive RR-TB cases had a median (IQR) age of 40 (31–52) years, 54.5% were male and 53.0% were referral cases from outside Bandung. Of those tested, 118 had a failed test and 339 (10.5%, Fig 2) patients were identified with RR-TB. Those diagnosed with RR-TB had a mean age of 38 years, 56.9% were male, over 2/3 were from outside Bandung and 21.5% were urban migrants, who had left their rural hometowns where they were registered (Table 1). Most (55.5%) had been referred because of failure of drug-sensitive TB treatment, almost all (97.1%) had DS-TB treatment history. Two hundred and eighty-eight (84.9%) started MDR-TB treatment at some point. Within the clinic system the step with the biggest loss to follow-up in the care cascade was between Xpert result and pre-treatment examination (48 of 339 [14.2%]; Fig 3).

**Table 1:** Characteristics of Xpert rifampicin-resistant TB patients (n = 339).

Characteristics	n (%)*
Male	193 (56.9)
Age (mean ± SD)	38.2 ± 12.5
HIV positive**	5 (1.5)
Residence	
Outside Bandung	231 (68.1)
Bandung	108 (31.9)
Employment status	
Unemployed	83 (24.5)
Retired, housewife, student	124 (36.6)
Employed	132 (38.9)
Monthly household income	
Do not have income	175 (51.6)
< 74 USD^±^	35 (10.3)
74–148 USD	80 (23.6)
>148 USD	49 (14.5)
Presumptive MDR-TB criteria	
Failure on HRZE/HRZES	188 (55.5)
Treatment default	29 (8.6)
Relapse	113 (33.3)
Contact with MDR-patient	4 (1.2)
History/current use of insufficient treatment regimen	5 (1.5)
History of drug-sensitive TB treatment	
None	7 (2.1)
Once	124 (36.6)
Twice	132 (38.9)
> 2 times	76 (22.4)
Sought care from private practitioners	
Yes	45 (13.3)
No	294 (86.7)
Source of referral for Xpert test	
Distric public hospital	139 (41.0)
Private hospital	34 (10.0)
Private practitioners	11 (3.3)
Primary Health Centre	131 (38.6)
Lung Clinic	24 (7.1)

*unless otherwise stated.

**Data were missing for HIV status (8.6%).

^±^1USD = 13,300 Indonesian Rupiah (IDR).

### Time to diagnosis and treatment of rifampicin-resistant TB

**Fig 4:**
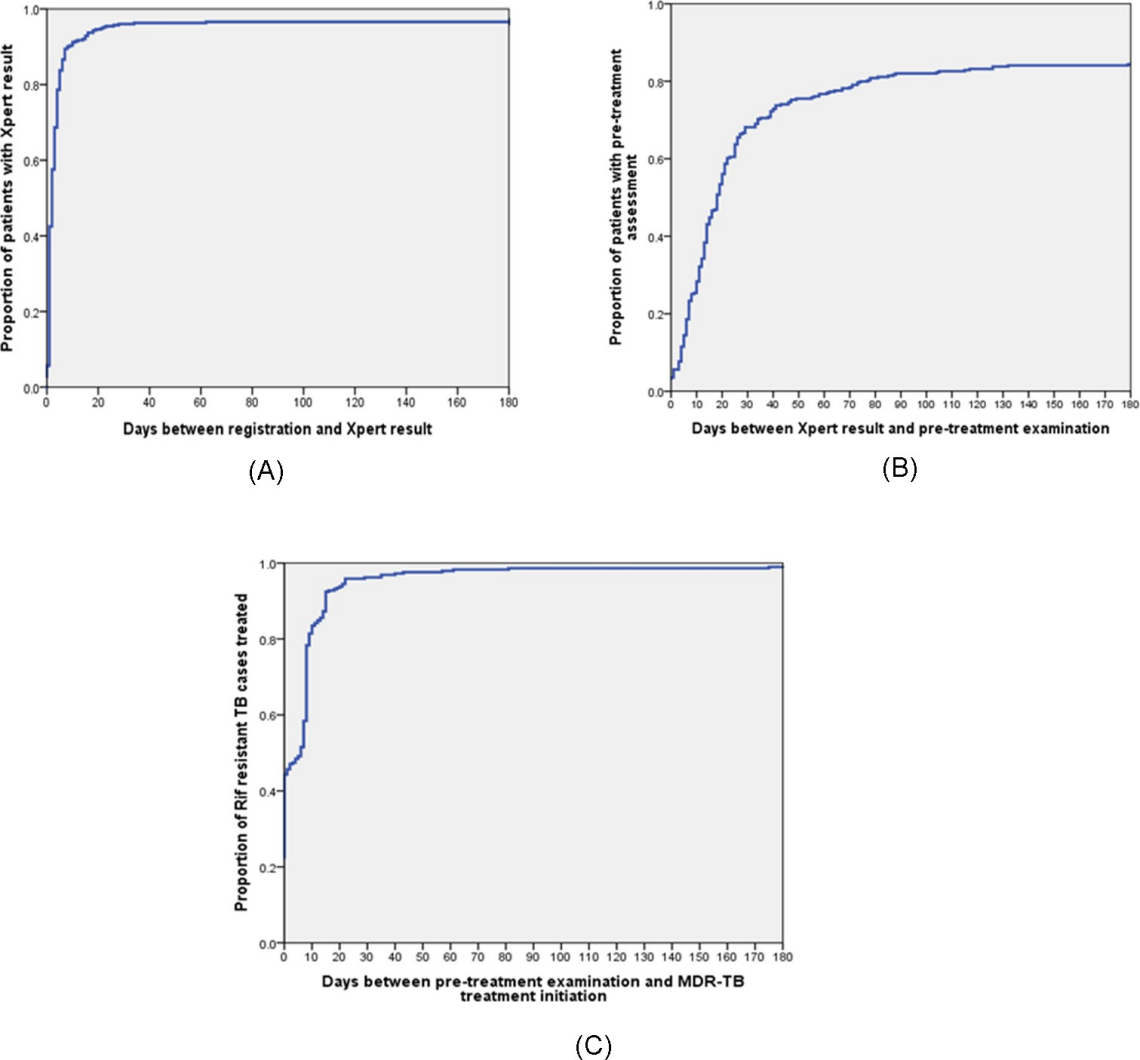
Kaplan Meier curves for time between specific cascade of care steps. These figures show Kaplan-Meier time-to-event graphs for specific cascade of care steps between registration and Xpert result (n = 351) (A), Xpert result and pre-treatment examination (n = 339) (B), and pre-treatment examination and MDR-TB treatment initiation (n = 291) (C).

The median time to diagnosis of RR-TB for those who did attend the clinic was 14 days (IQR 7–29; Fig 3), noting that there were no data available on those who were referred but did not attend the PMDT clinic. It took a median of 10 days (IQR 5–24) from having the smear microscopy result at a primary or secondary healthcare provider (HCP) and a patient’s visit to the PMDT clinic. The median duration between submitting sputum and getting the Xpert result was two days (IQR 1–4; Fig 4A). Gender, age, income, presumptive RR-TB criteria, and frequency of previous TB treatment were not associated with diagnostic delay (Table 2). However, patients who came from outside Bandung were more likely to have a diagnostic delay (odds ratio 2.7; 95% CI: 1.5–5.2).

**Table 2:** Multivariable analysis of factors associated with diagnostic and treatment delay of rifampicin-resistant TB patients.

Variable	Diagnostic delay (n = 201)					Treatment delay (n = 339)				
	n (%)	OR	95% CI	AOR	95% CI	n (%)	OR	95% CI	AOR	95% CI
Male	57 (49.6%)	1.2	0.7–2.2	1.1	0.6–1.9	138 (71.5%)	0.8	0.5–1.4	0.8	0.5–1.3
Age (years)	38.5±13.5	1.0	0.9–1.0	1.0	0.9–1.0	38.6±12.5	1.0	0.9–1.0	1.0	0.9–1.0
Residence:										
Bandung	19 (30.6%)	Reference		Reference		70 (64.8%)	Reference		Reference	
Outside Bandung	76 (54.7%)	2.7	1.4–5.1**	2.7	1.5–5.2**	177 (76.6%)	1.8	1.1–2.9	2.0	1.2–3.4**
Monthly household income:										
Do not have income	44 (46.8%)	Reference		Reference			Reference		Reference	
< 74 USD	15 (48.4%)	1.1	0.5–2.4	0.9	0.4–2.1	24 (68.6%)	0.8	0.4–1.8	0.8	0.3–1.8
74–148 USD	21 (44.7%)	0.9	0.5–1.9	0.8	0.4–1.8	54 (67.5%)	0.8	0.4–1.4	0.7	0.4–1.3
>148 USD	15 (51.7%)	1.2	0.5–2.8	1.3	0.5–3.1	42 (85.7%)	2.3	0.9–5.4	2.5	1.0–6.0
Presumptive MDR-TB criteria:										
Treatment failure	65 (48.5%)	1.2	0.6–2.1	1.2	0.7–2.2	138 (75%)	1.3	0.8–2.0	1.3	0.8–2.1
Other criteria*	30 (44.8%)	Reference		Reference		109 (70.3%)	Reference		Reference	
Frequency of previous TB treatment:										
≤ 2 times	68 (44.7%)	Reference		Reference		188 (71.5%)	Reference		Reference	
> 2 times	27 (55.1%)	1.5	0.8–2.9	1.3	0.7–2.6	59 (77.6%)	1.4	0.8–2.5	1.3	0.7–2.5

OR: odds ratio; AOR: adjusted odds ratio.

*Other criteria: default, relapse, contact history with MDR-TB patients, TB-HIV co-infection and history/current use of insufficient treatment regimen.

**p<0.05.

The median time to start second-line treatment was 25 days (IQR 15–67). After having an Xpert result, it took a median of 18 days (IQR 8–47) for patients to visit the PMDT clinic for pre-treatment examination (Fig 4B), and a median of 6 days between pre-treatment assessment and start of second-line treatment (IQR 0–8; Fig 4C). Patients who came from outside Bandung were more likely to have treatment delay (odds ratio 2.0; 95%CI: 1.2–3.4; Table 2). Overall, the median time between TB diagnosis and start of second-line treatment was 41 days (IQR 29–70), and 104 (51.7%) of 201 RR-TB patients with identifiable referral date experienced both diagnostic and treatment delays.

### Phenotypic drug susceptibility testing results and treatment adjustment

Sputum culture results were available for 243 patients (71.7%) and phenotypic DST results for 162 (47.8%) of 339 patients with Xpert-positive RR-TB. Phenotypic DST identified 107 of 339 patients with Xpert RR-TB as having MDR-TB (31.6%), 28 patients (8.3%) as having pre-XDR-TB- mostly with fluoroquinolone resistance, 4 (1.2%) as having XDR-TB (Table 3), and 23 (6.8%) as not having MDR-TB. No phenotypic DST was available for 177 patients (52.1%) who had a negative culture, a non-tuberculous mycobacteria (NTM) as culture result, a contaminated culture, or no culture result. Focusing on those with a DST result who started MDR-TB treatment (n = 158), we confirmed 6 (3.8%) as RIF mono-resistance, 105 (66.5%) as MDR-TB, 30 (18.9%) as preXDR- or XDR-TB (Fig 2). 129 (79.6%) of 135 patients with confirmed MDR-, pre-XDR- or XDR-TB phenotypically received an appropriate treatment regimen at some point (Fig 2). For 17 RR-TB patients with phenotypically confirmed RIF-susceptibility, MDR-TB treatment was continued without further adjustment.

**Table 3:** Sputum culture and phenotypic drug resistance of 243 Xpert rifampicin-resistant TB patients.

Test	n (%)
Sputum culture (n = 243)	
Positive	162 (66.7)
Negative	57 (23.5)
Non-tuberculous mycobacteria	16 (6.6)
Contaminated	8 (3.3)
Individual drug-resistance (n = 162)	
Rifampicin	145(89.5)
Isoniazid	128 (79.0)
Ethambutol	95 (58.6)
Streptomycin	67 (41.4)
Kanamycin^#^	8 (4.9)
Amikacin^#^	4 (2.5)
Ofloxacin^#^	33 (20.4)
Drug resistance by definition (n = 162)	
MDR-TB*	107 (66.0)
Pre-XDR TB**	28 (17.3)
Pre-XDR with injectables resistance	2 (1.2)
Pre-XDR with fluoroquinolone resistance	26 (16.1)
XDR-TB***	4 (2.5)
RIF monoresistance	6 (3.7)
INH monoresistance	3 (1.9)
Monoresistance^a^	1 (0.6)
Polyresistance^b^	1 (0.6)
RIF-sensitive	12 (7.4)

^#^Missing data for two patients.

*Multidrug-resistant TB (MDR-TB): resistant to at least isoniazid and rifampicin.

**Pre-XDR TB: resistant to isoniazid and rifampicin and either a fluoroquinolone and second-line anti-TB injectable drugs, but not both.

***Extensively drug-resistant TB (XDR-TB): resistant to isoniazid and rifampicin (as well as any fluoroquinolone and at least one of three second-line anti-TB injectable drugs (amikacin, kanamycin and capreomycin.

^a^Monoresistance: resistance to one first-line anti-TB drug only.

^b^Polyresistance: resistance to more than one first-line anti-TB drug, other than both isoniazid and rifampicin.[8]

### Estimation of the number of pre-XDR and XDR-TB patients

We estimated the number of patients with pre-XDR- and XDR-TB who were likely to have been missed by the system. Assuming that proportion with Xpert RIF resistance among 3415 MDR-TB suspects entering the system was the same among those who were tested as those not tested or had a failed test, overall 374 referred patients were estimated to have had Xpert RIF resistance. Based on simple extrapolation from the phenotypic DST results in those with a test result, it can be estimated that 65 of these patients had pre-XDR TB and 9 had XDR-TB. Therefore, we estimated that almost a fifth, 74 out of 374 patients with RIF resistance entering the system would have had pre-XDR-TB or XDR-TB, but that only 30 (40.6%) were identified and 24 (32.5%) were commenced on an appropriate regimen.

## Discussion

This study has identified specific losses and delays with respect to diagnosis and treatment initiation of RR-TB in Indonesia. Of 3415 patients referred with presumptive RR-TB over a 2-year period, almost 10% (n = 339) were diagnosed with Xpert RR-TB. For this group it took a median of two weeks to obtain an Xpert result and an additional 25 days until start of second-line treatment, with 14% of patients lost between diagnosis and MDR-TB treatment initiation. Patients who came from outside the city were more likely to have diagnostic and treatment delay. Just over half of those with Xpert-diagnosed RR-TB who started initial second-line treatment had a phenotypic DST result. Of those with DST results, 66% had MDR-TB and 20% had either pre-XDR or XDR-TB. If the phenotypic DST data are extrapolated to the whole study population of registered patients, then the system diagnosed only 41% of pre-XDR or XDR-TB patients and started only 33% on an appropriate treatment regimen. Together these data provide important insights into the challenges of diagnosing and treating DR-TB in this setting.

Currently, in West Java province, healthcare facilities equipped with Xpert are centralized in urban areas, which likely explains the poorer performance of the system for patients from outside the city. Our hospital is the tertiary level hospital in West Java, providing referral services for patients from lower-level healthcare providers in 18 regencies and 9 municipalities that serve a total population of 46.4 million. Among RR-TB patients from outside the city, one third were urban migrants. Previous studies in China have shown that urban migrants have higher TB rates, more drug resistance, and poorer treatment outcomes than urban residents [32, 33]. Several decentralized models of care for MDR-TB have been introduced to improve the linkage between diagnosis and treatment initiation especially for patients from rural areas [34, 35]. MDR-TB patients treated under community-based care involving healthcare workers and treatment supporters at sub-district or village level achieved equally high treatment success as those with hospital-based initiation [36].

Referral of patients to PMDT hospital for Xpert testing took a median of 10 days, in line with a study in India which showed a median delay of 8 days [37]. In a study in South Africa, diagnostic delay was caused by patient delays, failure to make a diagnosis at the first visit, and patients visiting public and private health care settings at the same time [38]. Negative perceptions of the public sector (over-burdened, long waiting times, negative attitudes of health care staff, lack of privacy), lack of money for transport, and lack of awareness that TB can relapse have been identified as barriers to prompt diagnosis [39]. In addition, major financial and social support constrains are underlying barriers [40].

The pre-treatment loss (one in seven) was surprisingly high in this study, despite the use of Xpert testing. Clinic staff reported that they called these patients multiple times, but that no home visits were done. Other studies have identified health system issues as important with respect to drop out, including: poor recording of patient contact details [41]; poor follow-up due to overwhelmed healthcare workers; interruptions due to dissatisfaction with the service; work and family commitments; and test results not being ready at follow-up appointments [39, 42]. In our study, we had to retrieve Xpert RIF resistant results from the laboratory register and second-line treatment data from the clinic database. As has been reported for other settings, insufficient linkage of such data systems increases the risk that patients with DR-TB are not started on treatment or are started on inappropriate regimens [43].

There was a considerable delay (median of 25 days) between Xpert diagnosis of RIF resistance and start of second-line treatment, even longer than reported in a previous study in Indonesia (median 15 days) [30], but similar to studies from other settings [20, 43, 44]. A systematic review reported that time from DR-TB diagnosis to second line treatment was shorter with genotypic than with phenotypic DST (38 versus 108 days) [45]. Rapid treatment initiation following RR-TB diagnosis is essential for TB control as it optimizes treatment outcomes, minimises disease transmission, and reduces further drug resistance [46, 47]. Optimal linkage to second line treatment will be similarly important.

Our data are in line with other studies showing that implementation of Xpert increases detection of RR-TB but that the clinical impact of this rapid DST is modest in weak health systems marked by gaps between diagnosis and treatment initiation [43, 48–50]. The roll-out of Xpert as universal DST has been hampered by high costs of scale-up, infrastructural problems such as lack of uninterrupted power supply, maintenance and technical support, interruptions in the supply chain, and lack of monitoring and supervision [7, 21].

It is of concern that less than half of patients with Xpert RR-TB had a phenotypic DST result to guide treatment, especially for those patients with pre-XDR or XDR-TB. Failure to detect additional drug resistance results in inadequate treatment regimens that contribute to poor treatment outcomes and higher mortality [51]. Culture is not routinely performed in Indonesia; only a fraction of patients put on TB treatment have their sputum cultured. Failed culture may be due to inability to collect sputum or sputum of good quality [52, 53], sample processing [54], contamination, or other laboratory issues. In our study, we found that 17 of RR-TB patients had RIF susceptibility on phenotypic DST, indicating the need for repeat DST testing and/or post-hoc *rpoB* gene sequencing to reconcile the discordant results [55].

Currently, sputum smear microscopy is the frontline diagnostic tool in primary healthcare settings in many high burden TB countries [56]. Xpert as a more sensitive diagnostic tool for TB remains underutilised in many countries, including Indonesia [57]. Widespread uptake of Xpert into primary or secondary level healthcare facilities as done in South Africa [58] will facilitate RR-TB detection, but phenotypic DST remains important for all patients with positive results. As shown elsewhere [59–61], Xpert testing and phenotypic DST show some discordance underlining the need for systematic algorithms to bring resolution. Furthermore, several diagnostic tools are in the pipeline to provide better detection of drug resistant TB such as the GeneXpert OMNI, the Xpert Ultra and the Xpert XDR [62, 63].

Some patients may receive treatment outside the NTP, especially from private providers, but they are unlikely to receive an appropriate treatment regimen [64]. Therefore, engagement of private practitioners by the NTP in Indonesia is a high priority area. A study from India showed that only an estimated 14% RR-TB cases were appropriately diagnosed and completed MDR-TB therapy in the public sector, with many patients evaluated at diagnostic facilities but few adequately diagnosed as MDR-TB [25]. Previous studies have also revealed critical gaps along drug-resistant TB cascade of care [49, 65]. In a South African study, an estimated 88% of RR-TB cases who accessed the diagnostic service received a diagnosis and 56% underwent second-line treatment [26].

Our study has several limitations. First, our findings may not be fully representative of Indonesia as data collection was only done in one tertiary hospital. Second, no data were available to accurately estimate the population prevalence of RR-TB to enable estimation of the proportion of RR-TB cases in the community entering the system. Third, this study did not take into account the proportion of presumptive RR-TB cases who were failed to reach the Xpert facility, which is one of the weakest links identified elsewhere across the TB cascade of care [66]. Fourth, we did not gather more explanatory data with respect to possible patient-related delays, such as for those who were registered at the PMDT clinic but failed to have an Xpert test, missed follow-up appointments, seeking care at other healthcare facilities, and socio-economic obstacles. Fifth, as noted above, we did not resolve discordant results between Xpert and phenotypic DST. Sixth, we did not evaluate MDR-TB treatment outcomes, which is key for a complete cascade of care analysis but beyond the scope of this study. Finally, since our study relied heavily on routine data, some information on baseline characteristics were missing.

To our best knowledge, this is the first study to report on losses and delays in diagnosis and treatment in RR-TB patients under programmatic conditions in Indonesia. Many patients with RR-TB were either lost to the system, experienced diagnostic or treatment delay, did not have DST results, or were not commenced on an appropriate treatment regimen. Decentralization of Xpert testing to district hospitals or primary healthcare facilities and private providers will be an important step to scale-up Xpert, and more will have to be done to enhance quality of culture-based DST and RR-TB management in Indonesia.

## Supporting information

S1 File DRTB dataset. (XLSX)

S1 Table Definitions of nine groups at risk of multidrug-resistant TB in line with Indonesian guidelines for programmatic management of drug-resistant TB. (DOCX)
