# Supplementary material for: Evaluation of Xpert MTB-RIF guided diagnosis and treatment of rifampicin-resistant tuberculosis in Indonesia: A retrospective cohort study
Source: PLoS One. 2019 Feb 28;14(2):e0213017. doi: 10.1371/journal.pone.0213017 (PMC6394995; doi:10.1371/journal.pone.0213017)
Supplement: S1 Table — (DOCX) [file pone.0213017.s002.docx]

**S1 Table. Definitions of nine groups at risk of multidrug-resistant TB in line with Indonesian guidelines for programmatic management of drug-resistant TB.**

| Risk category | Definition |
| --- | --- |
| 1. Chronic cases, mostly patients who failed first-line TB re-treatment | Patients who are still sputum smear-positive at the end of first-line TB re-treatment (Category 2) |
| 1. Patients on first-line TB re-treatment without smear conversion | Patients on first-line TB re-treatment (Category 2) who test smear-positive after three months of treatment |
| 1. Patients with previous TB treatment outside of national program | Patients who received any tipe of TB treatment outside of the national programme, e.g. non-DOTS or private clinics |
| 1. Patients who failed first-line TB treatment | Patients who are still sputum smear-positive at the end of first line TB treatment (Category 1) |
| 1. Patients on first-line TB treatment without smear conversion | Patients on first-line TB treatment (Category 1) who test smear-positive after three months of treatment |
| 1. Relapse cases | Patients whose most recent treatment outcome (Category 1 or 2) was ‘cured’ or ‘treatment completed’ and return with symptoms of TB |
| 1. Patients returning after loss to follow-up | Patients who interrupted any type of TB treatment for two or more consecutive months and return with symptoms of TB |
| 1. Close contacts of MDR-TB patients | People living in the same household or spending many hours a day in the same indoor living space of an MDR-TB patient and who show symptoms of TB |
| 1. Patients co-infected with HIV and TB | Patients who tested positive for HIV and TB with diagnostic tests |
| *Abbreviations: TB, tuberculosis; DOTS, direct observed treatment strategy; MDR-TB, multidrug-resistant tuberculosis; HIV, human immunodeficiency virus.* | |
